# Predicting Liver Fibrosis in the Hepatitis C Population: Concordance Analysis Between Noninvasive Scoring Systems and Percutaneous Liver Biopsy

**DOI:** 10.7759/cureus.10376

**Published:** 2020-09-11

**Authors:** Pradeep Kumar Mada, Matthew E Malus, Daniel Alexander Saldaña Koppel, Sharon Adley, Maureen Moore, Mohammed J Alam, Mark Feldman

**Affiliations:** 1 Infectious Diseases, Louisiana State University Health Sciences Center, Shreveport, USA; 2 Internal Medicine, Texas Health Presbyterian Hospital, Dallas, USA; 3 Internal Medicine, Louisiana State University Health Sciences Center, Shreveport, USA

**Keywords:** hepatitis c, liver biopsy, fibrosis-4 (fib-4) score, aspartate aminotransferase-to-platelet ratio index (apri score), aspartate aminotransferase-to-alanine aminotransferase ratio (aar)

## Abstract

Background

Due to the slow progression of many chronic liver diseases, including hepatitis C, it is not practical or safe to monitor disease progression by serial liver biopsies. Noninvasive laboratory scoring systems based on routine laboratory tests are appealing surrogate markers of liver fibrosis for the staging and monitoring of chronic liver diseases such as hepatitis C.

Methods

We explored the accuracy of three scoring systems: the fibrosis-4 score (FIB-4), the aspartate aminotransferase to platelet ratio index (APRI score), and the aspartate aminotransferase to alanine aminotransferase ratio (AAR) in 496 patients with chronic hepatitis C virus (HCV) infection who had undergone percutaneous liver biopsy at a viral hepatitis clinic in Shreveport, Louisiana.

Results

For FIB-4, the area under the receiver operating characteristic curve (AUROC) for hepatic fibrosis stages ≥ 1, ≥ 2, ≥ 3, and 4 (cirrhosis) ranged from 0.74 (95% CI, 0.678 - 0.802) to 0.802 (95% CI, 0.751 - 0.854). At a cutoff value of 1.45, FIB-4 was 82% sensitive for advanced fibrosis or cirrhosis (stage 3 or 4) but was only 58% specific for these findings. Increasing the FIB-4 cutoff value to 3.25 reduced the sensitivity for detecting advanced fibrosis or cirrhosis to 39%, but this higher cutoff was 92% specific for these findings. Corresponding AUROCs for the APRI and AAR scores were inferior to FIB-4.

Conclusion

The FIB-4 index outperformed APRI and AAR in our HCV infected population in predicting severe fibrosis or cirrhosis.

## Introduction

Hepatitis C is a treatable chronic liver disease that if left untreated or if unsuccessfully treated may lead to hepatic fibrosis and eventually to irreversible cirrhosis with complications and the need for a liver transplant [1]. Liver histology is the gold standard for the diagnosis and staging of hepatic fibrosis and cirrhosis in chronic hepatitis C [2]. However, a liver biopsy to determine histopathology can have procedure-related complications and is also limited by the need for expertise in performing the biopsy, cost, observer interpretation, sampling error, and patient unwillingness. Conventional blood tests (serum aspartate aminotransferase (AST), serum alanine aminotransferase ratio (ALT), and platelet count) have been used to try to estimate the degree of hepatic fibrosis, which, if highly predictive of fibrosis, could serve as a surrogate for liver biopsy [3-5]. We, therefore, performed a retrospective study in nearly 500 patients with chronic hepatitis C subjected to percutaneous liver biopsy at one institution, comparing the ability of three laboratory-based indices to predict the degree of hepatic fibrosis. Indices examined were the fibrosis-4 score (FIB-4), the aspartate aminotransferase to platelet ratio index (APRI score), and the aspartate aminotransferase to alanine aminotransferase ratio (AAR).

## Materials and methods

Study population

We performed a retrospective chart review in a viral hepatitis clinic in Shreveport, Louisiana. Hospital electronic health records were screened for the diagnosis of chronic hepatitis C by specific International Classification of Diseases 10th Revision Clinical Modification (ICD-10-CM) code B18.2 [6]. Patients were included if they were greater than 18 years old, had chronic hepatitis C, were seen in the clinic between November 1, 2014, and December 31, 2017, were treatment naïve, had had laboratory testing done for serum aspartate aminotransferase (AST), serum alanine transaminase (ALT), and platelet count, and had had a percutaneous liver biopsy during this period. A total of 496 patients were included. We recorded the patients’ age at the time of liver biopsy, their gender, hepatitis C virus (HCV) viral load and genotype, liver biopsy results as well as serum AST and ALT levels and platelet count near the time of liver biopsy. All patients tested negative for human immunodeficiency virus (HIV) and hepatitis B virus infections. Laboratory tests were typically done within the one week period prior to liver biopsy.

Noninvasive Scoring Systems

FIB-4 score: [Age (years) x serum AST (U/L)] / [Platelets (10^9^/L) x √ serum ALT (U/L)].

APRI score: [(serum AST (U/L) / (gender-related upper limit of normal serum AST (U/L)] / [Platelet count (10^9^/L)].

AAR: serum AST (U/L) / serum ALT (U/L).

Liver Biopsy

All percutaneous liver biopsies were performed by two senior gastroenterologists. The biopsy samples were at least 25 mm long and a minimum of 10 portal tracts was included in each specimen to improve diagnostic accuracy. All liver tissue samples were analyzed twice by one senior hepatopathologist. The METAVIR scoring system was used to assess the extent of hepatic fibrosis. This fibrosis staging score represents the amount of fibrosis, scored from F0 to F4 (Stage F0 = no fibrosis, Stage F1 = mild fibrosis, Stage F2 = significant fibrosis, Stage F3 = severe fibrosis, and Stage F4 = cirrhosis) [7-8].

Statistical analysis

Data were entered in a Microsoft Excel (Microsoft Corporation, Redmond, Washington) sheet, coded to de-identify patients, and analyzed in the Statistical Package for the Social Sciences (SPSS) v22.0 (IBM Corp., Armonk, New York). Percentages were calculated for categorical variables. Means and standard error of means (SEM) were determined for continuous variables. Analysis of variance (ANOVA) was performed to compare mean laboratory values at different grades of fibrosis. Receiver operating characteristic (ROC) curves were drawn and the area under the receiver operating characteristic curve (AUROC) was estimated to compare the diagnostic efficiency of the three noninvasive scores: FIB-4, APRI, and AAR.

## Results

The majority of the patients (71%) were middle-aged (36-59 years) and just over half of them were women (Table 1). The hepatic fibrosis grades for the 496 study patients are shown in Table 2, with only 42 patients having no fibrosis (F0) and 74 having cirrhosis (F4). The F1 and F2 stages were the largest categories (n = 142 and n = 144, respectively). Mean serum AST and ALT increased, and platelet counts decreased, as the extent of hepatic fibrosis increased. Likewise, FIB-4 and AAR (but not APRI) increased stepwise as the fibrosis grade increased, whereas mean APRI and AAR did not increase until F3 and F4 fibrosis was reached (Table 2). There were no significant differences in calculated non-invasive scores between women and men.

**Table 1:** Baseline characteristics of the study population (n=496) HCV: hepatitis C virus, SEM: standard error of the mean ^a^ 326 (66%) were genotype 1A and 84 (17%) were genotype 1B

Characteristic		Mean
Age in mean (±SEM)		53 ± 0.4
Gender	Female	273 (55%)
	Male	223 (45%)
Race	Black	252 (50.8%)
	White	241 (48.6%)
	Hispanic	2 (0.4%)
	Arabic	1 (0.2%)
HCV genotype	1	410 (83%)^a^
	2	47 (9%)
	3	33 (7%)
	4	6 (1%)

**Table 2:** Relationship between noninvasive markers (mean±SEM) with hepatic fibrosis grade on liver biopsy in 496 patients with chronic hepatitis C *P < 0.0001by ANOVA AAR, aspartate aminotransferase to alanine aminotransferase ratio; ALT, alanine aminotransferase; ANOVA, analysis of variance; APRI, aspartate aminotransferase to platelet ratio index; AST, aspartate aminotransferase; F0, no fibrosis, F1, mild fibrosis, F2, significant fibrosis, F3, severe fibrosis and F4, cirrhosis; FIB-4, fibrosis-4 score; P, probability; SEM, standard error of the mean ^a^ mean±SEM in U/L ^b^ mean±SEM in 109/L ^c^ mean±SEM

Test	Serum AST^a^ *	Serum ALT^a^ *	Platelet count^b ^*	FIB-4^c^ *	APRI^c^	AAR^c^ *
F0 (N = 42)	39±4.1	56±6.8	251±7.4	1.0±0.08	1.0±0.21	0.75±0.03
F1 (N = 142)	42±2.3	56±2.9	225±5.8	1.6±0.13	0.9±0.08	0.79±0.02
F2 (N = 144)	55±3	79±4.4	207±5.8	1.9±0.13	1.0±0.15	0.80±0.02
F3 (N = 94)	86±9.8	98±8.7	179±6.2	3.0±0.2	1.1±0.1	0.87±0.02
F4 (N = 74)	100±8.4	110±9.3	150±7.3	4.2±0.35	1.2±0.11	0.95±0.03

**Figure 1:**
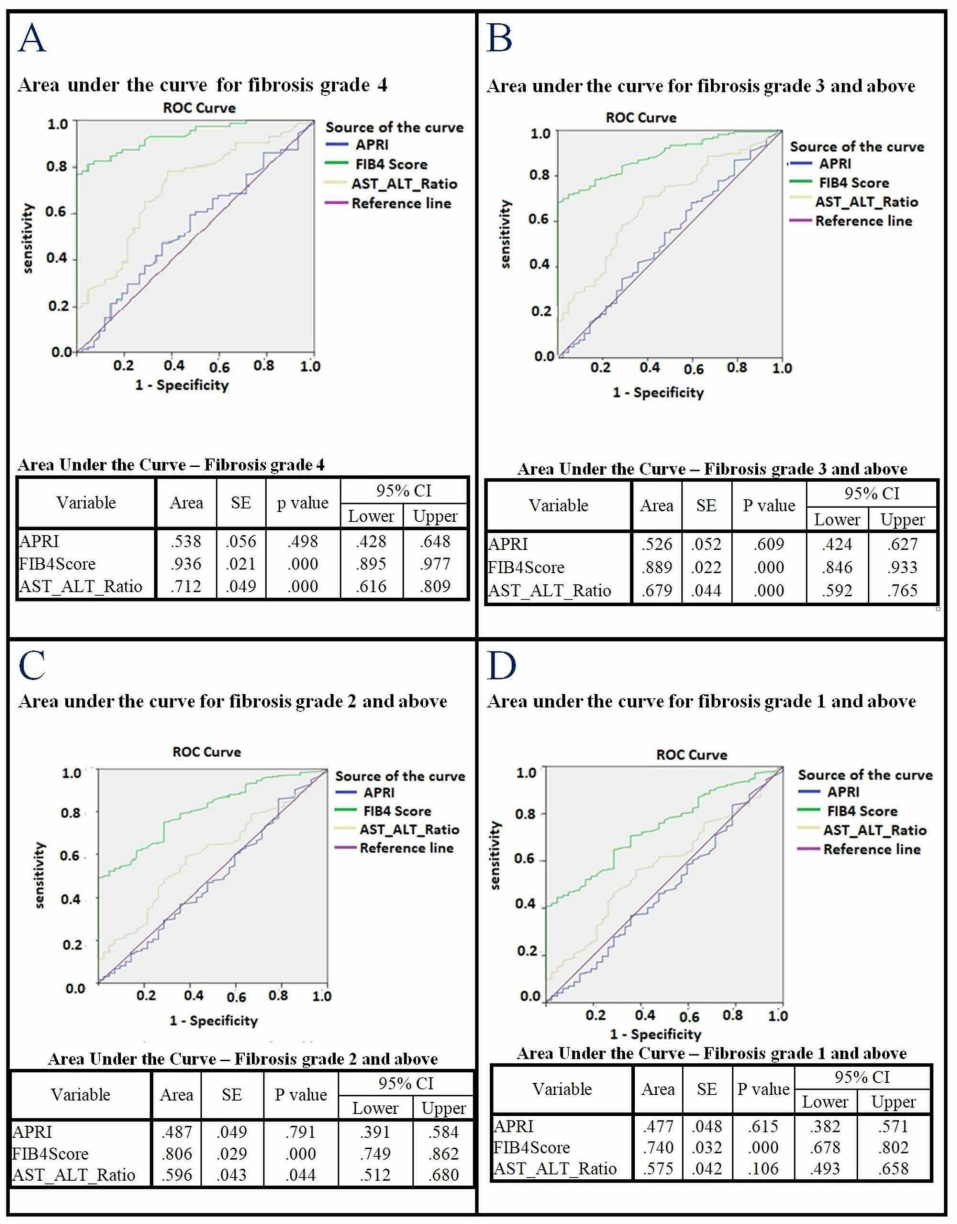
FIB-4, AAR, and APRI scores in predicting various degrees of hepatic fibrosis The FIB-4 score consistently had the largest area under the curve for predicting any grade of fibrosis or cirrhosis. FIB-4, fibrosis-4 score; AAR, aspartate aminotransferase to alanine aminotransferase ratio; APRI, aspartate aminotransferase to platelet ratio index

**Figure 2:**
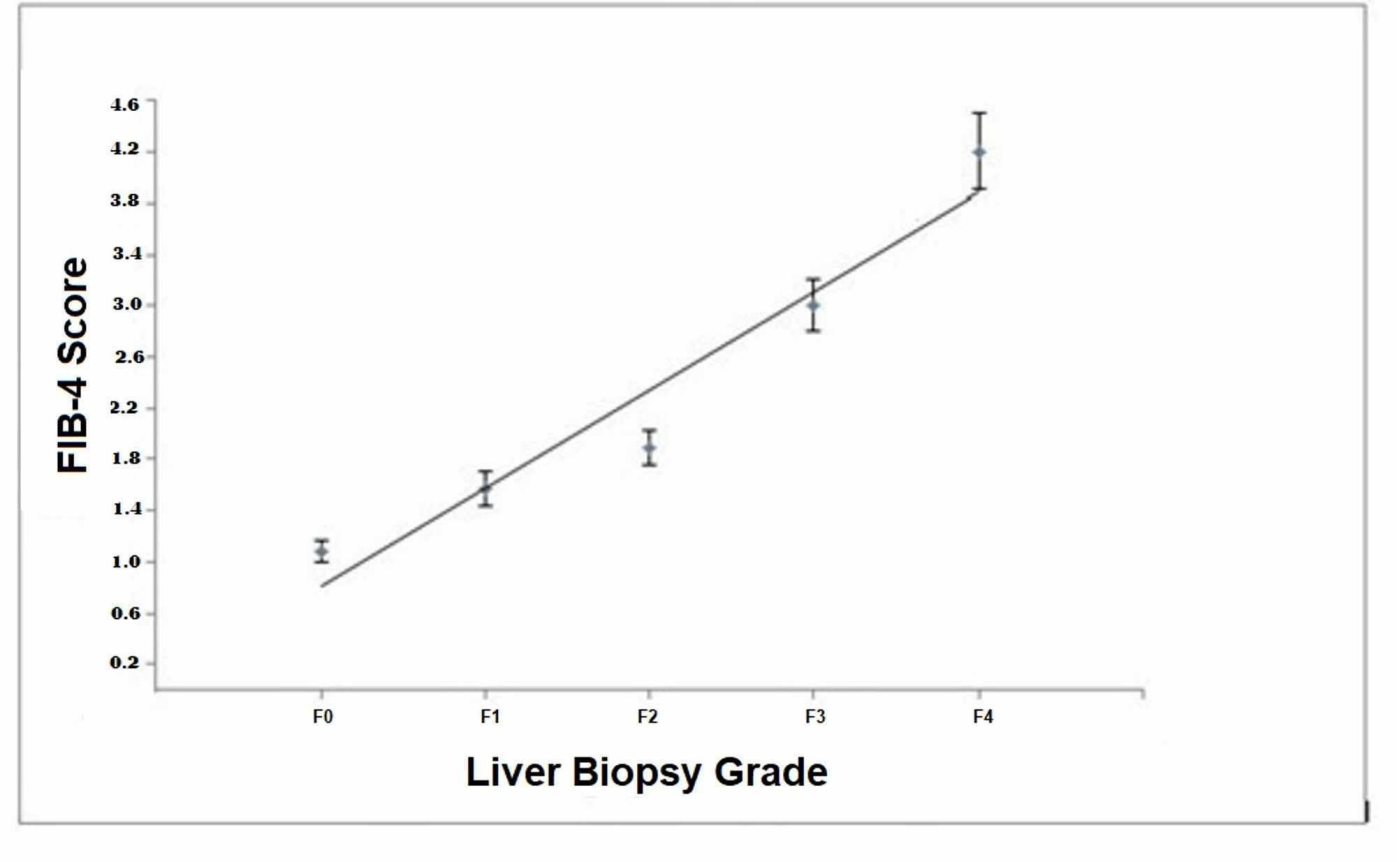
Relationship of mean (±SEM) FIB-4 scores with fibrosis grade on percutaneous liver biopsy in 496 patients with chronic hepatitis C Mean FIB-4 scores showed a significant correlation with fibrosis grade (p < 0.001); The number of patients for each liver biopsy grade are shown in Table 1. FIB-4, fibrosis-4 score

Figure 1A-1D displays the three non-invasive marker scores in predicting various degrees of hepatic fibrosis. In each case, FIB-4 outperformed AAR and APRI. The FIB-4 score had the highest area under the curve for predicting cirrhosis (F4) with an area under the curve (AUC) of .802 (95% CI: .751 - .854) (Figure 1A). FIB-4 outperformed AAR and APRI in predicting severe fibrosis or cirrhosis (F3 or F4) (Figure 1B), moderate to severe fibrosis or cirrhosis (F2 to F4; Figure 1C), or any grade of fibrosis or cirrhosis (F1 to F4; Figure 1D), with AUC values ranging from .732 to .788. Mean FIB-4 scores showed a good correlation with fibrosis grade (R = 0.97; p < 0.001; Figure 2).

A FIB-4 index of ≥ 3.25 had a 72% positive predictive value, 92% specificity, and diagnostic accuracy of 0.74 in predicting severe fibrosis or cirrhosis (F3 or F4). A FIB-4 index of ≤ 1.45 had an 86% negative predictive value and 82% sensitivity in excluding severe fibrosis or cirrhosis (F3-F4). The diagnostic accuracy of a FIB-4 cutoff of ≥3.25 was 0.74. The Youden statistic is used for the evaluation of the overall discriminative power of a diagnostic procedure and for comparison of the test with other available tests. A FIB-4 score cutoff of ≤ 1.45 had the highest Youden index, the maximum potential effectiveness of a biomarker (0.4), followed by a FIB-4 score cutoff of ≥ 3.25 (0.31). The diagnostic odds ratio (DOR) of a test is a ratio of the odds of positivity in subjects with the disease to the odds in subjects without the disease. A FIB-4 score cutoff of ≥3.25 had the highest DOR (7.75) followed by the FIB-4 score cutoff of ≤ 1.45 (6.24) (Table 3).

**Table 3:** Test characteristics of each noninvasive score in prediction of severe fibrosis or cirrhosis AAR, aspartate aminotransferase to alanine aminotransferase ratio; APRI, aspartate aminotransferase to platelet ratio index; DOR, diagnostic odds ratio; FIB-4, fibrosis-4 score; LR-, likelihood ratio negative; LR+, likelihood ratio positive; NPV, negative predictive value; PPV, positive predictive value ^a^ percentage

Score	Cutoff	Sensitivity^a^	Specificity^a^	PPV^a^	NPV^a^	LR +	LR -	Youden index	DOR	Diagnostic accuracy
FIB-4	≤ 1.45	82	58	50	86	1.94	0.31	0.4	6.24	0.66
FIB-4	≥ 3.25	39	92	72	75	5.12	0.66	0.31	7.75	0.74
APRI	≤ 0.7	52	59	39	71	1.26	0.82	0.1	1.52	0.57
APRI	≥ 1	36	73	41	69	1.34	0.88	0.09	1.54	0.61
AAR	≤ 1	31	86	53	71	2.18	0.8	0.17	2.71	0.67

## Discussion

In 2006, Sterling et al. developed FIB-4, an index that might predict liver fibrosis, in 832 patients with HIV/HCV co-infection from 95 centers across 19 countries. The authors concluded that the AUROC was 0.765 for differentiation between Ishak stages 0-3 (no or mild fibrosis) and 4-6 (marked fibrosis/bridging or cirrhosis) using the suggested FIB-4 cut-off values of ≤ 1.45 (negative predictive value (NPV) 90%, sensitivity 70%) to ≥ 3.25 (positive predictive value (PPV) 65%, specificity 97%) [3]. The authors estimated for this HIV/HCV cohort that liver biopsy could have been replaced by the FIB-4 index with 86% accuracy. FIB-4 was further validated by Kim et al. in 2010 (AUROC for prediction of significant (F≥2) and severe (F ≥3) fibrosis, and cirrhosis (F4) were 0.865, 0.910, and 0.926, respectively) [4]. Our study results using a different liver biopsy scoring system (METAVIR) were in fairly close agreement with the study of Sterling et al. [3], with 86% NPV and a sensitivity of 82% for a ≤ 1.45 cutoff and a PPV of 72% and specificity of 92% for ≥ a 3.45 cutoff. The positive likelihood ratio was highest (5.12) with a FIB-4 cutoff of ≥ 3.25, and the negative likelihood ratio was lowest (0.31) for a FIB-4 cutoff of ≤ 1.45.

Our study is unique in that it represents a single-center US study with nearly 500 patients, which included mostly HCV genotype 1 and without HIV or hepatitis B coinfection. A single-center study helped minimize variations in inter-observer biopsy interpretation and avoided differences in processing in different labs. Other studies performed in other countries and a multi-center study in the USA have yielded similar results of AUROC for the FIB-4 index [9-14].

In 2003, Wai et al. developed the APRI score to predict significant fibrosis and cirrhosis in patients with chronic HCV. The authors concluded that the AUC for predicting significant fibrosis was 0.88 and was 0.94 for cirrhosis. The cut-off values in the Wai study were ≤ 0.50 and ≥ 1.50 for predicting the absence or presence of significant fibrosis/cirrhosis (Ishak score ≥3), respectively [15]. Lin et al. performed a meta-analysis in 2011 where an APRI cutoff of 0.7 had 77% sensitivity and 72% specificity for significant fibrosis and a cutoff of 1.0 had 61% sensitivity and 64% specificity for severe fibrosis [16]. Our study showed that an APRI score of 1.0 had a PPV of only 41% and a specificity of 73%, inferior to FIB-4.

Williams et al. observed that both AST and ALT levels rose with the progression of liver damage, specifically in patients with chronic hepatitis, and a ratio of AAR >1.0 would typically suggest cirrhosis with 100% specificity and a PPV in distinguishing cirrhotic from non-cirrhotic patients, with a 53% sensitivity and 81% NPV [17-18]. Our study showed that an AAR score of < 1.0 had an NPV of 71% to exclude severe fibrosis (F3-F4), a sensitivity of only 31%, a positive predictive value of only 53%, and a specificity of 86%, also inferior to FIB-4 using the AUC. Determining fibrosis severity is critical in chronic liver disease, as it predicts long-term clinical outcomes and death in HCV [19]. In contrast with the above studies, including ours, some authors concluded that noninvasive markers are not a reliable tool to predict liver fibrosis. Parkes et al. reviewed 10 different serum markers of hepatic fibrosis in chronic hepatitis C. Only 35% of patients had fibrosis adequately ruled in or ruled out by these panels, and the stage of fibrosis could not be adequately determined [20]. The calculation of FIB-4 can simply be done from routine labs that can be reassessed every accurately [21-22].

## Conclusions

The FIB-4 score was the better predictor across all the grades. The AAR ratio was the next best predictor, and the APRI score was inferior as compared to the other two. When the scores were compared within each grade, it was found that the efficiency increases as the grade increases in all three scores. In summary, the FIB-4 score had better diagnostic accuracy than AAR and APRI. These non-invasive scores, particularly FIB-4, do fairly well in ruling out rather than ruling in advanced disease, having higher negative predictive values than positive predictive values.

## References

[1] Poynard T, Mathurin P, Lai C-L (2003). A comparison of fibrosis progression in chronic liver diseases. J Hepatol.

[2] Bravo AA., Sheth SG, Chopra S (2001). Liver biopsy. N Engl J Med.

[3] Sterling RK, Lissen E, Clumeck N (2006). Development of a simple noninvasive index to predict significant fibrosis in patients with HIV/HCV coinfection. Hepatology.

[4] Kim BK, Kim DY, Park JY (2010). Validation of FIB-4 and comparison with other simple noninvasive indices for predicting liver fibrosis and cirrhosis in hepatitis B virus-infected patients. Liver Int.

[5] Siddiqui MS, Yamada G, Vuppalanchi R (2019). Diagnostic accuracy of noninvasive fibrosis models to detect change in fibrosis stage. Clin Gastroenterol Hepatol.

[6] (2016). ICD-10-CM Code B18.2. Chronic viral hepatitis C. https://icd.codes/icd10cm/B182.

[7] The French METAVIR Cooperative Study Group, Bedossa P (1994). Intraobserver and interobserver variations in liver biopsy interpretation in patients with chronic hepatitis C. Hepatology.

[8] Bedossa P, Poynard T (1996). An algorithm for the grading of activity in chronic hepatitis C. Hepatology.

[9] Cordie A, Salama A, El-Sharkawy M (2018). Comparing the efficiency of Fib-4, Egy-score, APRI, and GUCI in liver fibrosis staging in Egyptians with chronic hepatitis C. J Med Virol.

[10] Ferenci P, Aires R, Beavers KL (2014). Predictive value of FIB-4 and APRI versus METAVIR on sustained virologic response in genotype 1 hepatitis C patients. Hepatol Int.

[11] Holmberg SD, Lu M, Rupp LB (2013). Noninvasive serum fibrosis markers for screening and staging chronic hepatitis c virus patients in a large US cohort. Clin Infect Dis.

[12] Stibbe KJM, Verveer C, Francke J (2011). Comparison of non-invasive assessment to diagnose liver fibrosis in chronic hepatitis B and C patients. Scand J Gastroenterol.

[13] Wang H-W, Peng C-Y, Lai H-C (2017). New noninvasive index for predicting liver fibrosis in Asian patients with chronic viral hepatitis. Sci Rep.

[14] Sirli R, Sporea I, Bota S (2010). A comparative study of non-invasive methods for fibrosis assessment in chronic HCV infection. Hepat Mon.

[15] Wai C, Greenson JK, Fontana RJ, Kalbfleisch JD, Marrero JA, Conjeevaram HS, Lok A S-F (2003). A simple noninvasive index can predict both significant fibrosis and cirrhosis in patients with chronic hepatitis C. Hepatology.

[16] Lin Z-H, Xin Y-N, Dong Q-J (2011). Performance of the aspartate aminotransferase-to-platelet ratio index for the staging of hepatitis C-related fibrosis: an updated meta-analysis. Hepatology.

[17] Sheth SG, Flamm SL, Gordon FD, Chopra S (1998). AST/ALT ratio predicts cirrhosis in patients with chronic hepatitis c virus infection. Am J Gastroenterol.

[18] Williams ALB, Hoofnagle JH (1988). Ratio of serum aspartate to alanine aminotransferase in chronic hepatitis relationship to cirrhosis. Gastroenterology.

[19] Ishak K, Baptista A, Bianchi L (1995). Histological grading and staging of chronic hepatitis. J Hepatol.

[20] Parkes J, Guha IN, Roderick P, Rosenberg W (2006). Performance of serum marker panels for liver fibrosis in chronic hepatitis C. J Hepatol.

[21] Vallet-Pichard A, Mallet V, Nalpas B (2007). FIB- 4: an inexpensive and accurate marker of fibrosis in HCV infection. comparison with liver biopsy and fibrotest. Hepatology.

[22] Castéra L, Foucher J, Bernard P-H (2010). Pitfalls of liver stiffness measurement: a 5-year prospective study of 13,369 examinations. Hepatology.

